# Equity in health and healthcare in Malawi: analysis of trends

**DOI:** 10.1186/1471-2458-7-78

**Published:** 2007-05-15

**Authors:** Eyob Zere, Matshidiso Moeti, Joses Kirigia, Takondwa Mwase, Edward Kataika

**Affiliations:** 1 World Health Organization, P.O.Box 30390, Lilongwe, Malawi; 2 World Health Organization, Regional Office for Africa, B.P. 6, Brazzaville, Congo; 3 PHR *plus*, P.O.Box 30846, Lilongwe, Malawi; 4 Ministry of Health, P.O.Box 30377, Lilongwe, Malawi

## Abstract

**Background:**

Growing scientific evidence points to the pervasiveness of inequities in health and health care and the persistence of the *inverse care law*, that is the availability of good quality healthcare seems to be inversely related to the need for it in developing countries. Achievement of the Millennium Development Goals is likely to be compromised if inequities in health/healthcare are not properly addressed.

**Objective:**

This study attempts to assess trends in inequities in selected indicators of health status and health service utilization in Malawi using data from the Demographic and Health Surveys of 1992, 2000 and 2004.

**Methods:**

Data from Demographic and Health Surveys of 1992, 2000 and 2004 are analysed for inequities in health/healthcare using quintile ratios and concentration curves/indices.

**Results:**

Overall, the findings indicate that in most of the selected indicators there are pro-rich inequities and that they have been widening during the period under consideration. Furthermore, vertical inequities are observed in the use of interventions (treatment of diarrhoea, ARI among under-five children), in that the non-poor who experience less burden from these diseases receive more of the treatment/interventions, whereas the poor who have a greater proportion of the disease burden use less of the interventions. It is also observed that the publicly provided services for some of the selected interventions (e.g. child delivery) benefit the non-poor more than the poor.

**Conclusion:**

The widening trend in inequities, in particular healthcare utilization for proven cost-effective interventions is likely to jeopardize the achievement of the Millennium Development Goals and other national and regional targets. To counteract the inequities it is recommended that coverage in poor communities be increased through appropriate targeting mechanisms and effective service delivery strategies. There is also a need for studies to identify which service delivery mechanisms are effective in the Malawian context.

## Background

There has been increased attention to issues of equity in health and healthcare with the renewed commitment of governments and international organizations to improve the health status of the poor and marginalized [1,2]. Equity is one of the basic principles of the Primary Health Care approach [3] and features implicitly or explicitly in the health policies of most countries [4].

Growing scientific evidence points to the pervasiveness of inequities in health and healthcare both between and within countries at different stages of development [5]. Despite achievements in the second half of the 20^th ^Century in improving life expectancy and child survival, inequities in health have persisted and in some cases have even widened [6].

It is now a well established fact that the poor and marginalized segments of society have a greater need for health care than their rich counterparts. However, access to healthcare still follows the *inverse care law *– the availability of good quality healthcare seems to be inversely related to the need for it [7].

Despite the commitment of governments to pursue pro-poor health policies and interventions vigorously, in sub-Saharan Africa the level of inequity in health status and access to basic health care interventions remains high. Benefit-incidence studies in a number of African countries have unequivocally shown that government expenditures on health tend to benefit the richest of society in absolute terms. On average the richest 20% receive more than twice the financial benefit than the poorest 20% of the population from overall government health spending [8].

Monitoring trends in equity in health and access to essential health interventions is important in order to target scarce public resources to those who have more needs, i.e. the poor. Poor countries in sub-Saharan Africa face many constraints in collecting and processing relevant information for gauging trends in equity. This, however, should not be a cause for inaction. It is possible, even in the poorest countries with the least resources, to do much more with the existing data and resources than what is being done currently [9]. Many countries in Africa have conducted various studies such as the demographic and health surveys (DHS) and household income and expenditure surveys. The availability of data for different time intervals makes it possible to review changes in equity in health and healthcare.

The objective of this report is to assess the trends in equity in Malawi for the various indicators of health and healthcare using data from the Malawi Demographic and Health Surveys of 1992, 2000 and 2004.

## Brief country profile

Malawi, a landlocked country in Southern/Central Africa, has an area of about 118,484 square kilometers, one-third of which is made up by Lake Malawi [10]. Based on its Human Development Index (HDI) of 0.404, the country ranks 165^th ^out of 177 countries and is classified as one of the low human development countries. Furthermore, the HDI has declined from its level of 0.412 in 1995 to a level of 0.404 in 2003 [11], indicating a drop in society's welfare.

The per capita GDP in 2003 was US$ 156 with an annual growth rate of 0.9% during the period 1990–2003. The GDP per capita for Malawi is much lower than the average values for low income and sub-Saharan African countries.

According to the 2004/2005 Integrated Household Survey (IHS), about 52% of Malawi's population is classified as poor, i.e. below a national poverty line of MWK 16, 165 per person per year – the equivalent of US$ 147 at that time. The median per capita income of the richest decile is about eight times that of the poorest decile [12].

Health and development indicators of Malawi are those typical of other low-income countries in sub-Saharan Africa, as depicted in Table 1.

**Table 1:** Malawi: Health and development indicators

**Characteristic**	**Value**
Total population (millions) (2003)	12.3
Annual population growth rate (%) (1994–2004)	2.4
Life expectancy at birth (male/female) (years)	41/41
Infant mortality rate (per 1000 live births) (2004)	76
Under-five mortality rate (per 1000 live births) (2004)	133
Total fertility rate (2004)	6.0
Maternal mortality ratio (per 100,000 live births)	984
Stunting in under-five children (%) (2004)	47.8
Adult (15–49 years) HIV prevalence rate (%) (2003)	11.8
Prevalence of tuberculosis (per 100,000) (2003)	551
Reported malaria rate (per 1,000) (2002)	240
Per capita total expenditure on health, 2002 at average exchange rate, US$ (2003)	13
Official development assistance per capita (US$) (2003)	45.4
Physicians per 100,000 population (2004)	2.0
Nurses per 100,000 population (2004)	59

Sources: Ref. [11, 13-16]

During the period 1990–2004, infant and under-five mortality rates have declined by an annual average of 5%. This is a significant decline compared to that in many countries in the region and exceeds the average annual reduction rate of about 4.3% required to achieve the targets of the Millennium Development Goal related to reducing child mortality by two-thirds between 1990 and 2015 (MDG 4). However, population averages do not always represent the reality. The average annual reduction rates for the poorest 20% of the population for infant and under-five mortality rates in Malawi are in the order of 2.2% and 2.7% respectively- much lower than the population average. Hence, although it appears potentially feasible to achieve the targets of MDG 4 with the current population average annual reduction rates, disaggregation by wealth quintile indicates that the poorest 20% are unlikely to achieve it.

The greatest proportion of the disease burden is composed of infectious and parasitic diseases and nutritional disorders. However, like most developing countries undergoing demographic and epidemiological transition, non-communicable diseases are also on the increase – thus posing an additional problem to a health system that is grappling with communicable diseases that sometimes assume epidemic proportions.

The per capita total expenditure on health is one of the lowest in sub-Saharan Africa and is critically short of the US$ 34 recommended by the WHO Commission on Macroeconomics and Health to provide a basic package of services [17]. The total expenditure on health amounts to about 9.8% of the GDP. Government expenditure on health constitutes only 41% of the total health expenditure. Furthermore, expenditure on health constitutes only 9.7% of total government expenditure. This is far below the Abuja target – a resolution by the African Heads of State to allocate 15% of the national budget to health.

The country's health service delivery system is four-tiered, consisting of community, primary, secondary and tertiary care levels [18]. At the community level, service is provided through health surveillance assistants. The focus is on preventive interventions. Primary care is delivered through clinics and health centres. District and central hospitals provide secondary and tertiary care services respectively. The private not-for-profit sector plays a significant role in service provision.

In order to address the enormous health problems effectively with very limited resources, the country has designed an essential healthcare package (EHP) as part of its health Sector-wide Approach (SWAp) adopted in 2004. The EHP being delivered at community, primary and secondary levels of the healthcare delivery system is provided free of charge. The EHP addresses the most common causes of morbidity and mortality and focuses mainly on health problems that disproportionately affect the poor [18].

## Equity: concept and measurement

Health-related equity may be viewed from three perspectives: (i) equity in health; (ii) equity in health service delivery; and (iii) equity in health financing. Operational definitions of the first two are given below, as they constitute the focus of this study.

Equity in health is defined as minimizing avoidable inequalities in health and its determinants – including but not limited to healthcare – between groups of people who have different levels of underlying social advantage or privilege [19]. Inequities exist when there are disparities in health and its determinants that are deemed to be avoidable, unfair and unjust [20]. Hence not all health inequalities between population groups are regarded as inequities. Inequities in health specifically refer to disparities between groups of people related to their social position as measured by such characteristics as income/wealth, occupation, education, geographic location, gender and race/ethnicity [9]. Health inequalities due to inevitable and unavoidable conditions (e.g. biological/genetic variations) do not constitute inequities.

The focus of equity in healthcare provision is to ensure that all people have access to a minimum standard of healthcare according to need and not any other criteria, such as ability to pay. In this case, equity may therefore be defined as *equal access for equal need*, where access refers to the absence of barriers – mainly geographical and financial barriers; and need refers to the *capacity to benefit *or *severity of illness*. Equity in service provision takes two forms: horizontal equity and vertical equity. While horizontal equity implies equal treatment for equal need, vertical equity implies that individuals with unequal needs should be treated unequally according to their differential need.

The Measurement of equity in health and healthcare entails three important steps: (i) classifying people by socio-economic status; (ii) measuring health status/healthcare; and (iii) quantifying the degree of inequality.

Measuring household economic status in developing countries is a difficult exercise. This is because data on two frequently used indicators of wealth – household income and expenditure – are often scarce and unreliable [21]. In developing countries, studies have shown a close relationship between asset ownership and consumption expenditure [22] and that household assets are a good indicator of the long-run economic status of households [21]. Asset indices are established to classify households into wealth quantiles (e.g. quintiles, deciles) using the method of Principal Components Analysis (PCA). Analysis of Demographic and health surveys of many countries conducted by the World Bank demonstrates the use of PCA to compute asset indices from data on durable consumer goods (e.g. ownership of radio, television *etc*.), housing quality (e.g. floor type), water and sanitary facilities and other amenities [21]. This categorization of households into wealth quintiles is used in this report to analyze inequities.

The next step in assessing equity is to devise appropriate measures of health and healthcare. Having decided on the attribute of health/healthcare to be compared among individuals/population groups, it is then important to find an appropriate technique to quantify the degree of the existing inequality. Several methods have been in use to date. Some have their origin in research on income inequality (e.g. Lorenz curve and the associated Gini coefficient) [23,24] or from modifications of these (e.g. concentration index) [25]. Other methods are based on measures of association (index of dissimilarity, slope index of inequality) [26]. This report is based on the measurement of inequities using the concentration index and corresponding concentration curve.

## Methodology

### Source of data

The information used in this study is based on findings from the Malawi Demographic and Health Surveys of 1992, 2000 and 2004.

### Data analysis

**Figure 1:**
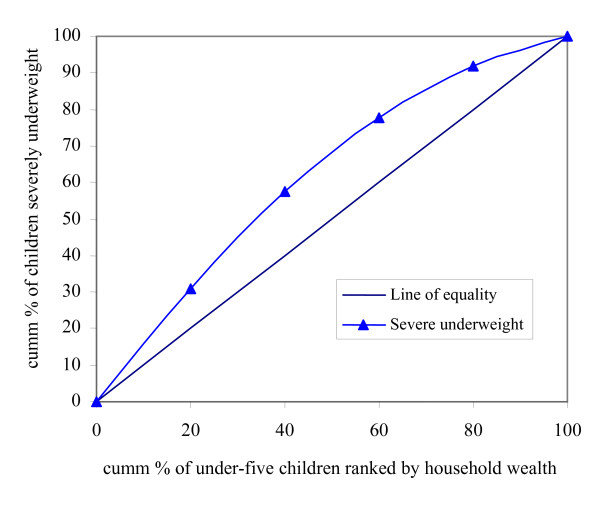
The concentration curve.

Inequities are represented by concentration curves that are relatively easier to understand compared to the concentration indices. The concentration curve plots the cumulative proportion of the individuals under consideration ranked by wealth against the cumulative proportion of the health/healthcare variable (e.g. stunting, under-five mortality rate, use of modern contraception *etc*.) being measured. To demonstrate the use of the concentration curve, the case of underweight (low weight-for-age) in under-five year old children is presented in Figure 1.

If there is no wealth-related inequality in the rate of severe underweight, the concentration curve would coincide with the diagonal line (line of equality). This implies that there are no inequities in severe underweight. However, if severe underweight has disproportionately higher prevalence among the poor, the concentration curve lies above the line of equality. The above example reveals that the poor are more likely to experience a greater burden of severe underweight in association with their socio-economic disadvantage (pro-rich inequity).

If the health indicator under consideration is an undesirable outcome such as severe underweight as in the above example, a concentration curve that lies above the line of equality signifies inequity disfavouring the poor and is bad from the equity point of view. If the indicator being considered is a desirable one (e.g. immunization coverage), a concentration curve that lies above the diagonal (line of equality) shows inequity favouring the poor – a situation that is desirable from the equity point of view. A point worthy of note is that the degree of inequity becomes more when the concentration curve is further from the line of equality.

In this study, the concentration curves for the different indicators of health/healthcare from the Malawi Demographic and Health Surveys of 1992, 2000 and 2004 are presented in the same figure so as to observe changes in inequities very easily.

The concentration index that is computed from the concentration curve assumes values between -1 and +1. Its value is negative when the concentration curve is above the diagonal and positive when the curve is below the diagonal. In the absence of inequities (the concentration curve coinciding with the diagonal), the value of the concentration index is zero.

From grouped data, the concentration index (C) is computed in a spreadsheet programme using the following formula [27]:

*C *= (*p*_1_*L*_2 _- *p*_2_*L*_1_) + (*p*_2_*L*_3 _- *p*_3_*L*_2_) + ... + (*p*_*T*-1_*L*_*T *_- *p*_*T*_*L*_*T*-1_),

Where *p *is the cumulative percent of the sample ranked by economic status, *L(p) *is the corresponding concentration curve ordinate and *T *is the number of socioeconomic groups. To test for the statistical significance of the concentration index, standard errors can be computed using the formula given in Kakwani *et al *[28].

## Results

This section presents the findings categorized into two groups: (i) indicators of health status; and (ii) indicators of health service use.

### Health status

Indicators of health status employed in this study include: infant mortality rate (IMR), under-five mortality (U5MR), under-five child malnutrition (represented by stunting – low height-for-age and underweight), prevalence of diarrhoea and acute respiratory infections (ARI), total fertility rate (TFR) and low body mass index (BMI) in women, which is an indicator of adult undernutrition. A summary of the distribution of the indicators is depicted in Table 2 below.

**Table 2:** Selected health status indicators

	1992		2000		2004	
	
Indicator	Population average	Quintile ratio (poor/rich)	Population average	Quintile ratio (poor/rich)	Population average^2^	Quintile ratio (poor/rich)^1^
Infant mortality rate	136	1.33	112	1.52	76	1.65
Under-five mortality rate	240	1.47	203	1.55	133	1.65
Children stunted (%)	49	1.53	49	1.72	47.8	1.68
Children underweight	35.3	2.17	25.5	2.55	22	2.25
Low mother's BMI	9.7	2.35	8.8	1.73	9.2	1.69
Total fertility rate	6.7	1.18	6.3	1.48	6.0	1.73
Prevalence of diarrhoea in under-five children (%)	21.7	1.13	17.6	1.36	22.3	1.46
Prevalence of acute respiratory infection in under-five children (%)	14.4	1.26	26.7	1.53	18.8	1.71

Sources: [*14*, *29–30*]; ^1 ^*own calculation*

As can be observed from Table 2, the population averages of indicators such as infant and under-five mortality showed significant improvement while in others there was little or no improvement. The quintile ratios indicate the presence of inequalities in all indicators that favour the rich. For most of the indicators (namely infant mortality rate, under-five mortality rate, total fertility rate, prevalence of ARI and diarrhoea in under-five children) widening of inequalities between the two extreme wealth quintiles (poorest 20 % and richest 20%) was observed. For example, while there was 33% more infant mortality in the poorest quintile as compared to the richest one in 1992, the excess mortality in the poorest quintile increased to 52% in 2000 and 65% in 2004. Thus even if there were slight improvements in the population averages, the improvements accrued more to the non-poor. A caveat is, however, in order here. The quintile ratios compare only the two extreme wealth quintiles (quintiles 1 and 5) and therefore disregard the situation of the three middle quintiles (quintiles 2, 3, and 4). Hence, the information provided does not give an overall measure of inequities in the entire population. It is for this reason that a summary measure – the concentration index/curve is needed.

**Figure 2:**
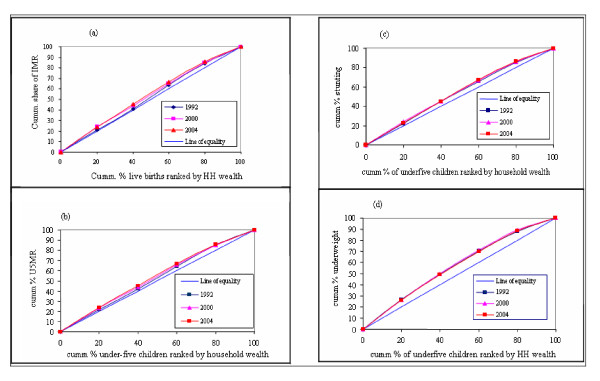
Concentration curves for selected health status indicators in children. (a) Infant mortality rate. (b) Under-five mortality. (c) Stunting. (d) Underweight.

As can be seen from Figure 2(a and b), there was an increase in the levels of pro-rich inequity in infant and under-five mortality rates in 2004 compared with the base period 1992. This implies that the burden of infant and under-five mortality was getting disproportionately higher among children from the poor than the non-poor households. For IMR, it is observed that the curve for 1992 is below those of 2000 and 2004 and closer to the line of equality, although it is not dominant (at some point there is intersection of the two curves). When the concentration curve for one year does not dominate the other one, it is helpful to resort to the corresponding summary measure, the concentration index, in order to have a clearer picture of the inequity. The concentration index (*C*) for 1992 was -0.03448 (standard error [SE] = 0.0341). This has no statistical significance and implies that in 1992, there was no inequity in IMR. However, it increased to -0.0434 (*SE *= 0.0006) in 2000, which is statistically significant inequity that disadvantages the poor. The same trend was observed in under-five malnutrition (Figure 2c and 2d) as measured by the levels of underweight and stunting. Thus, the interventions that were designed during this period to improve child survival and nutritional status did not benefit children from poorest segments of society.

Pro-rich inequities in the prevalence of diarrhoea and ARI among under-five children widened during the period considered. The concentration indices for diarrhoea in 1992 and 2000 respectively were -0.026 (SE = 0.011) and -0.067 (SE = 0.011). These figures are significantly different from each other and unequivocally indicate a widening of the inequity favouring the non-poor. Similarly for ARI the concentration indices for 1992 and 2000 respectively were -0.043 (SE = 0.015) and -0.054 (SE = 0.009). These figures also demonstrate a significant increase in the burden of ARI among the poor.

**Figure 3:**
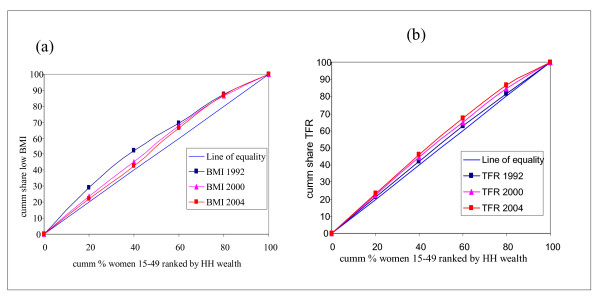
Concentration curves for selected health status indicators in women. (a) Low mother's body mass index (BMI<18.5). (b) Total fertility rate.

The indicators of women's health status in Table 2 tend to move in a different direction as can also be seen from Figure 3 below.

It can be observed from Panel 3a that the concentration curve for BMI in 2000 and 2004 was closer to the line of equality than it was in 1992. The pro-rich inequity in BMI in 1992 has diminished significantly over the years (The curve for 1992 was farther from the line of equality than the one for 2000; and the one for 2004 is the closest to the line of equality). The concentration index (C = 0.0802, SE = 0.0514) is also testimony to this. However, with respect to the TFR, a progressive increase in pro-rich inequities is observed – the TFR concentration curve for 2004 is farther from the line of equality than that of 2000 and of the base period, 1992.

### Health service use

Utilization rates of various mother and child health interventions are employed as indicators of health service use (Table 3).

**Table 3:** Selected indicators of health service use

	1992		2000		2004	
	
Indicator	Population average	Quintile ratio (poor/rich)	Population average	Quintile ratio (poor/rich)	Population average^2^	Quintile ratio (poor/rich)^1^
Immunization coverage (%)	81.8	0.82	70.1	0.80	64.4	0.67
ARI*: % medically seen if ill	53.7	0.76	26.7	0.39	19.6	0.67
ARI: treatment in public facility	36.5	0.73	18	0.58	N.A.	N.A
Diarrhoea: ORT** use	73.3	0.75	62.1	0.85	61.1	0.84
Diarrhoea: % seen if medically ill	49	0.76	28.3	0.79	36.4	0.85
Diarrhoea: % seen in public facility if ill	34.7	0.87	20.7	1.08	N.A.	N.A.
Antenatal visits to a medically trained person (doctor, nurse or nurse-midwife)	90.1	0.87	92.5	0.91	92.1	0.91
Delivery attendance by a medically trained person	54.9	0.57	55.6	0.52	56.1	0.55
Delivery: % of births at a public facility	41.2	0.58	40.2	0.56	41.9	0.61
Delivery: % of births at home	42.7	2.6	43.6	3.39	29.4	4.0

*Acute respiratory infection; ** *Oral rehydration therapy*; N.A. – *Data not available*

Source: [14, 29-30]; ^1 ^own calculation

As can be discerned from Table 3, in most of the service use indicators, there was no improvement in inequities that were in favour of the richest quintile. The degree of inequity increased substantially in the two ARI service indicators. On the other hand, a remarkable reduction in inequity was seen in ORT use and proportion treated for diarrhoea in public facility. It should also be noted from the above table that the inequity in the proportion of births taking place at home favours the poorest quintile. This implies that home delivery is mainly practiced by the poor compared to the non-poor. Furthermore, it is observed that the magnitude of the pro-poor inequity in home delivery has widened during the period under consideration, implying that the poor increasingly resorted to home delivery. As discussed earlier, comparison of two extreme quintiles (quintiles 1 and 5) excludes the situation of the middle quintiles. It is therefore essential to use the concentration curve and index to have a summary measure that takes into account the situation of all the five quintiles.

**Figure 4:**
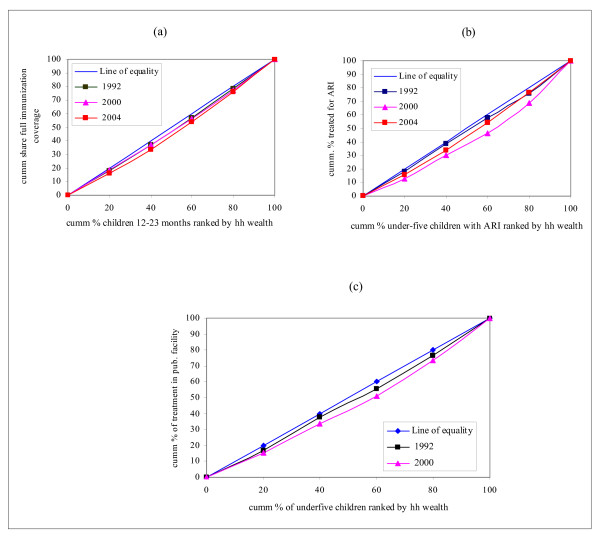
Concentration curves for selected health service use indicators in children: Immunization coverage and ARI treatment. (a) Immunization: basic full coverage. (b) ARI treatment. (c) ARI treatment in Public facility.

**Figure 5:**
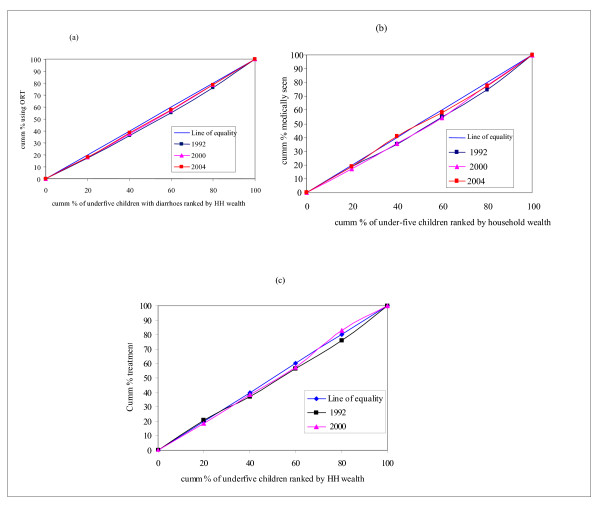
Concentration curves for selected health service use in children: ORT and treatment of diarrhoea. (a) ORT use among under-five children with diarrhea. (b) Diarrhoea – medically seen. (c) Diarrhoea treatment in public facility.

Figure 4(a and b) indicates that no improvements were seen in equity in the use of child health services related to immunization coverage and ARI treatment during the period under consideration. With respect to immunization coverage, the pro-rich inequity has increased. In 1992, there was no inequity in ARI treatment as observed from Figure 4(b) where the concentration curve is very close to the line of equality. However, caution should be exercised here. As it has been discussed in Section 5.1, pro-rich inequity is observed in the prevalence of ARI, that is, there is a high concentration of the ARI burden among children from the poorest households. If equity is to prevail, the principle of vertical equity (unequal treatment for unequal need) demands that those with greater need should receive more of the treatment. However, what is observed in the current case is that there is *equal treatment for unequal need *and clearly violates the requirements of vertical equity. Hence, there is inequity, as the poor who have a greater need for treatment as compared to the non-poor are not getting the treatment according to their need. Furthermore, Figure 4(b) shows that the concentration curve for 2000 has deviated from the line of equality significantly. This implies that use of public sector facilities has become more inequitable – the non-poor using the public sector healthcare resources more than the poor and out of proportion to their need. Other indicators of use of child health services include interventions related to the treatment of diarrhoea. Figure 5 below depicts this information.

Figure 5(a) on the use of ORT among those who reported diarrhoea in both time periods has a significant pro-rich orientation despite a slight reduction in the levels of inequity. No improvement was observed in equity in ORT use. If equity prevailed in the use of ORT, then the concentration curves should have been located above the line of equality. In other words, the poor should have used ORT more than the non-poor, as they bear the greatest burden of diarrhoeal disease as discussed in Section 5.1. The same trend was also observed in the case of those who sought medical attention for diarrhoea – the non-poor sought care more than the poorest. The *status quo *was maintained during the two time periods. There was no distinction between the poor and non-poor in terms of seeking care in a public facility. Caution should, however, be exercised here. The fact that there is no difference in use of public facilities for diarrhoea treatment among the poor and non-poor does not accord with the principle of vertical equity. Scarce public resources for the treatment of diarrhoea should be used more by the poorest who have more need for it as indicated by the relatively high prevalence of the condition among them.

**Figure 6:**
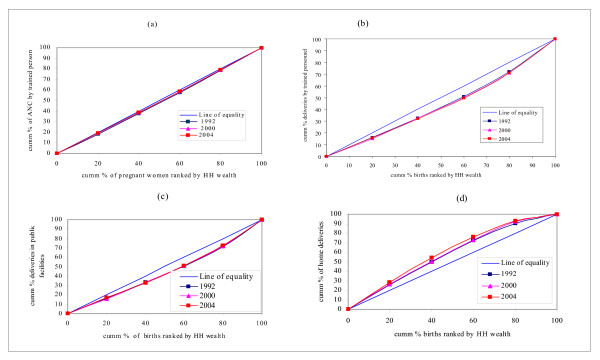
Concentration curves: antenatal care and child delivery services. (a) Antenatal care by medically trained personnel. (b) Delivery by medically trained personnel. (c) Delivery in public facilities. (d) Home delivery.

The other indicators of health service use employed to assess trends in equity are related to maternal health. These include use of antenatal and delivery services as depicted in Figure 6 below.

As can be observed from Figure 6(a), there was no significant difference in the state of pro-rich inequity in the use of antenatal services by medically trained personnel. The inequity in antenatal care use was, however, of a lesser magnitude compared to those of child delivery services. With respect to delivery services, the same trend of inequity was observed. As can be observed from Panel 6b, the degree of pro-rich inequity was more than that observed in antenatal care services, as the concentration curves are relatively far from the line of inequality.

Panel 6c clearly depicts the fact that publicly provided services for child delivery are utilized more by the non-poor. This implies that the non-poor benefit from public subsidies more than the poor – contrary to stated intentions of public policies. Panel 6d demonstrates that home-deliveries have pro-poor orientation. The poor utilize home delivery services excessively compared to the non-poor.

### Summary of findings

The findings described above are summarized in Table 4 using a framework for evaluating health equity changes [31].

**Table 4:** Summary of the changes in health and healthcare inequities

		**Relative gap**	
		
		**Narrowing**	**Widening/*status quo***
		*Best outcome:*	*Improvement for the better off but not for the poor:*
**Population average**	**Improving**	• Body mass index	• Infant mortality rate • Under-five mortality rate • Stunting • Underweight • Total fertility rate • Antenatal care • Child delivery • Child deliveries at home
		*Worsening with an element of protection for the poor:*	*Worst outcome:*
	**Worsening**	• ORT use	• Prevalence of diarrhoea among under-five children • Prevalence of ARI among under-five children • Immunization coverage • ARI treatment in public facility

## Discussion

This paper attempts to assess trends in inequities in selected health status and health services utilisation indicators in Malawi by using quintile ratios and concentration curves and indices. The analysis is based on data from the Demographic and Health Surveys of 1992, 2000 and 2004. This time period allows for analyzing trends in inequities of health indicators that often change gradually and over a longer period of time.

By and large, the findings indicate that in most of the selected indicators of health and healthcare, increases in pro-rich inequities have occurred. This is an undesirable trend in light of the government's explicit commitment to equity in health and healthcare and policy stances. Interventions intended to lessen inequities disfavouring the poor have not borne the expected results.

The quintile ratios for infant and under-five mortality rates indicate progressive inequities between the two extreme quintiles, i.e. wealth quintiles 1 and 5 during the period considered. This is also corroborated by the concentration curves in Figure 2, where the respective concentration curves for the year 2004 have moved further away from the line of equality. Thus, there was no improvement in inequities in these indicators and the improvement in the population averages was primarily due to marked improvements in the rates for the relatively wealthy segments of the population.

Although child mortality rates are influenced by a host of factors, many of which lie outside the health sector, they are often regarded as a proxy for overall disease conditions [17]. Infant and under-five mortality rates are closely related to economic growth and distribution of economic and social resources. Studies have shown that countries whose IMR rates are relatively lower enjoy better economic growth rates than those otherwise [17]. This significant correlation between child mortality rates and economic growth implies that, addressing inequities in infant and under-five mortality should be multi-sectoral and that beyond the biomedical solutions, there is a need to also address the underlying social determinants through concerted and complementary efforts of all sectors of the economy. This is also in line with the principles of the Primary Health Care strategy.

The main direct causes of mortality in under-five children are infectious diseases occurring because they were neither prevented (e.g. vaccine-preventable diseases) nor successfully treated (e.g. ARIs, diarrhoeal diseases) [32]. Diarrhoea, ARIs, measles, malaria and malnutrition account for at least 70% of childhood diseases [*32*]. The underlying causes are related to socio-economic factors. Thus, from the health sector's perspective, the immediate response to reducing infant and under-five mortality is improving access of the poor to preventive, curative and rehabilitative interventions that are geared towards addressing the major direct causes of childhood mortality. Improving coverage of the interventions through the Integrated Management of Childhood Illness (IMCI) programme may go a long way to bridge the inequity gaps, as 70% of the direct causes are related to the diseases and conditions covered in the IMCI strategy. In addition to improving access to health facilities, improving coverage of IMCI interventions also necessitates outreach services and an increase in community level activities [33]. Widening inequities may imply that the poor's access to the appropriate preventive, curative and rehabilitative interventions has not improved or has even declined.

With respect to child malnutrition (stunting and underweight), there has been an increase in inequities between 1992 and 2004. After a significant increase in inequities in 2000 from the 1992 levels, there was a marginal but statistically insignificant decline in 2004. Thus, no change was observed in the inequity levels in child malnutrition between 2000 and 2004.

According to the WHO cutoffs used to identify nutrition problems of public health significance, the population averages of both stunting and underweight in Malawi fall under the categories of severe stunting (cutoff ≥ 40%) and moderate underweight (cutoff 20–29%). Although the rate of stunting is high even in the non-poor wealth quintile (Quintile 5), there is a marked difference in comparison to that of the poorest quintile (Quintile 1). Stunting, which is an indicator of chronic malnutrition poses adverse long-term consequences on economic productivity. Hence, strategies aimed at reducing poverty and income inequalities need to also tackle the problem of stunting in the overall population and in particular among the poorest of society.

Inequities in total fertility rate (TFR) have been increasing progressively over the given period of time despite a marginal decrease in the population average. The average TFR for Malawi is one of the highest in countries of the Southern African Development Community. Widening inequities suggest that the marginal decline in TFR observed is due to a decrease in TFR among the non-poor. This implies that health sector-specific interventions to curb high fertility rates (e.g. uptake of contraceptives) are not benefiting the poor due to a number of reasons including problems of access and cultural barriers. High TFR has far-reaching effects in that it adversely affects child survival and household welfare particularly among the poor. It is therefore necessary that policies aimed at improving household welfare need to boost coverage of the poor with the available effective interventions. Furthermore, barriers to accessing those interventions need to be identified and addressed appropriately.

A remarkable achievement has been scored in low BMI (body mass index) of mothers, an indicator of maternal undernutrition. Pro-rich inequity that was observed during the earlier years (i.e. 1992 and 2000) was reversed in 2004. Hence there are no inequities in this indicator; maternal undernutrition does not vary systematically with socio-economic status. The DHS data also indicate that overweight and obesity are less of a problem among women from poor households [14].

The BMI, which is an indicator of chronic energy deficiency among adults, is less of a biomedical problem than it is socio-economic. It is influenced by a host of factors including household socio-economic status, household feeding patterns and seasonal factors [34]. It can therefore be discerned that improvement in those influencing factors among the poor was registered over the years, thus bridging the inequity gap. Reduction in the rate of low BMI in women is beneficial, as low pre-pregnancy BMI is an established risk factor for low birth weight [35], which in turn affects child survival negatively. It is therefore essential to identify the measures that effectively resulted in abolishing pro-rich inequities so as to replicate them in other related areas and avert any future relapses of inequity in BMI.

Inequities in the prevalence of diarrhoea and ARI among under-five children have also increased over the years significantly. These two conditions are among the major killers of children in sub-Saharan Africa and amenable to low-cost preventive and curative interventions. The fact that pro-rich inequities have widened may imply that environmental conditions (including biological, physical and social environments) that are necessary for the propagation of these diseases among the poor have been deteriorating. Many of the enabling factors for diarrhoeal diseases and ARIs are related to household and community-level socio-economic conditions. Therefore, preventing the disproportionately higher burden of diarrhoea among the poor needs a multi-sectoral strategy beyond the bounds of the health sector (e.g. provision of safe water supply; sanitation, decent housing *etc*).

The population average for immunization coverage in 2004 has declined by about 17 percentage points from the levels in 1992. Besides, the inequities in immunization coverage seem to have widened over the years implying that the immunization coverage among the poor has continuously declined. It is a well established fact that effective and equitable health systems are a pre-requisite for achieving the MDGs and other health goals [36]. Therefore, the current trend is likely to slow down or even reverse the achievement of the Millennium Development Goal aimed at reducing child mortality.

With respect to Diarrhoea and ARI interventions it has to be noted that an equitable condition demands that those with a higher burden of illness receive more of the treatment according to their need. Hence, the concentration curves should lie above the diagonal (line of equality). Equal use is not equitable in this case. As discussed earlier, diarrhoeal diseases and ARIs are among the major causes of morbidity and mortality among under-five children. It is therefore, necessary to identify the barriers to the utilization of these interventions by the poor so that the poor make use of these interventions more than the non-poor who have less need for it. The current situation of inequity may potentially affect progress towards the aforementioned MDG.

Although there is no inequity in antenatal care, delivery by medically trained personnel favours the non-poor. Moreover, delivery in public facilities is inequitable and to the advantage of the non-poor. This implies that the poor get less of the benefits of publicly financed/subsidized services, contrary to the government's policy objectives. Not unexpectedly, child delivery at home has a pro-poor orientation, which implies that the poor deliver at home proportionately more than the non-poor. The fact that government services are utilized more by the non-poor implies that the poor have a constrained access to child delivery services. This may be related to physical distance, low perceived quality or cultural barriers to name but a few. The definitive contributing factors should be identified by means of further studies. By and large, this trend is likely to jeopardize the pace of reducing maternal mortality and thereby achieving the MDG 5 target, that is reducing maternal mortality.

The *inverse equity hypothesis *proposed by victora *et al *[37] states that new interventions will initially benefit those of higher socio-economic status and only later do they reach the poor. This results in initial increase in inequity ratios for coverage, morbidity and mortality [36]. Policy makers should, therefore, take this phenomenon into account and counteract the widening of inequities through appropriate service delivery strategies. Increasing coverage in poor communities through targeting of those interventions that mainly benefit the poor as well as universal coverage of interventions that address conditions that significantly affect the poor is needed [38].

Overall pro-rich inequities in health and healthcare are widespread in Malawi and in some cases are widening despite the concerted efforts of government and its development partners. Improvements in population averages of the indicators should not be taken at face value, as the widening disparities imply that the MDG targets may be achieved by the non-poor, but the poor segments of society might not be able to reach them. The fact that the non-poor benefit more from the publicly provided services, which are highly subsidized, is also a point of concern that calls for effective means of targeting the scarce resources. Initiatives such as the sector-wide approach (SWAp) [39] and the design of essential healthcare package are not inherently equitable if not complemented with policies and strategies that uphold the principles of equity. It is therefore, important to assess interventions/initiatives not only in terms of their efficiency, but also their impact on equity through an appropriate equity gauge [40].

## Competing interests

The author(s) declare that they have no competing interests.

## Authors' contributions

EZ designed the study, performed the analysis and drafted the report; MM, JK, TM and EK participated in the write-up and revision of the manuscript.

## Pre-publication history

The pre-publication history for this paper can be accessed here:


